# Low Luminance Visual Acuity and Low Luminance Deficit in Choroideremia and *RPGR*-Associated Retinitis Pigmentosa

**DOI:** 10.1167/tvst.10.2.28

**Published:** 2021-02-18

**Authors:** Laura J. Wood, Jasleen K. Jolly, Amandeep S. Josan, Thomas M. W. Buckley, Robert E. MacLaren

**Affiliations:** 1 Nuffield Laboratory of Ophthalmology, Nuffield Department of Clinical Neurosciences, University of Oxford, Oxford, UK; 2 Oxford Eye Hospital, Oxford University Hospitals NHS Foundation Trust, Oxford, UK

**Keywords:** low luminance visual acuity, low luminance deficit, visual acuity, choroideremia, retinitis pigmentosa

## Abstract

**Introduction:**

Choroideremia and *RPGR*-associated retinitis pigmentosa (RP) are two distinct inherited rod-cone degenerations, where good visual acuity (VA) is maintained until late disease stages, limiting its usefulness as a disease marker. Low luminance VA and low luminance deficit (standard VA minus low luminance VA) may be more sensitive visual function measures.

**Methods:**

Standard VA was obtained using Early Treatment Diabetic Retinopathy Study letter charts (Precision Vision, Bloomington, IL, USA). Low luminance VA was assessed using a 2.0-log unit neutral density filter, with the same chart setup, without formal dark adaptation. Mean central retinal sensitivity was assessed using MAIA microperimetry (Centervue SpA, Padova, Italy). Optical coherence tomography imaging was attained with Heidelberg Eye Explorer software (Heidelberg Engineering, Heidelberg, Germany).

**Results:**

Twenty-four male participants with confirmed pathogenic *RPGR* mutations, 44 male participants with confirmed pathogenic *CHM* mutations, and 62 age-matched controls underwent clinical assessment prior to clinical trial recruitment. Low luminance VA was significantly reduced in both disease groups compared to controls. The low luminance deficit correlated with microperimetry retinal sensitivity and ellipsoid zone width. Eleven participants with moderate VA had poor low luminance VA (subsequently a large low luminance deficit), no detectable microperimetry sensitivity, and severely constricted ellipsoid zone widths.

**Conclusions:**

Low luminance VA and subsequently low luminance deficit are useful markers of central macular visual function in both choroideremia and *RPGR*-associated RP, when standard VA is preserved.

**Translational Relevance:**

Low luminance visual acuity and low luminance deficit are useful vision measures in two distinct rod-cone degenerations and may be useful in other retinal degenerations.

## Introduction

Inherited retinal degenerations are the leading cause of visual impairment in the working population.^1^ For many of these conditions, there are no treatments available, although this is changing with recent advancements in gene therapy. Therapies for conditions such as *RPE65* Leber congenital amaurosis, choroideremia, and *RPGR*-associated retinitis pigmentosa (RP) have shown promising results.^2^^–^^4^

Choroideremia and *RPGR*-associated RP are progressive X-linked inherited retinal degenerations. Choroideremia affects primarily the retinal pigment epithelium as well as the choroid and outer retina. The disease is due to pathogenic mutations in the *CHM* gene, affecting the function of the Rab escort protein, which is required to mediate photoreceptor and retinal pigment epithelium cell membrane transport.^5^ Patients present typically during the second decade of life with nyctalopia and progressive visual field loss, gradually progressing until severe visual impairment, often by the third or fourth decade.^6^

Pathogenic genetic variants in the retinitis pigmentosa GTPase regulator gene (*RPGR*) account for at least 70% of X-linked RP cases. Loss of the *RPGR* protein function critically affects protein transport across the photoreceptor outer and inner segments, leading to photoreceptor degeneration.^7^ Like in choroideremia, patients tend to present in the second decade with symptoms of nyctalopia and progressive visual field loss, as the condition typically manifests as a rod-cone degeneration.^8^ Severe visual impairment usually occurs by the fifth decade.^9^

Both choroideremia and *RPGR*-associated RP demonstrate variable disease progression.^10^^,^^11^ Good visual acuity (VA) is maintained until advanced stage disease,^7^^,^^12^ and therefore, VA alone is an insufficient visual functional assessment and a poor marker of vision-related performance in everyday life.^13^ Exploring alternative, reliable but more sensitive visual outcome measures for use in clinical trial is now paramount.

Low luminance VA (LLVA) involves standard VA testing in low-light conditions, often by adding a neutral density filter in front of the testing eye. It is a useful visual function marker in those with geographic atrophy and neovascular age-related macular degeneration following anti-vascular endothelial growth factor (VEGF) treatment.^14^^,^^15^ LLVA correlates significantly with symptoms of night vision loss,^16^^,^^17^ which is of great relevance in both choroideremia and *RPGR*-associated RP. Low luminance deficit (LLD) is frequently reported; this is the difference between standard VA and LLVA.^14^ A LLD greater than 13 Early Treatment Diabetic Retinopathy Study (ETDRS) letters suggests abnormality.^18^

Microperimetry is a fundus-tracked perimetry assessment of central retinal sensitivity. It enables accurate threshold sensitivity assessment at specific locations within the macula region. The test is most commonly performed in mesopic conditions and therefore is primarily a test of cone function, similar to VA.^19^ It is a repeatable and useful measure in choroideremia and *RPGR*-associated RP to map centripetal degeneration.^6^^,^^20^ Despite its effectiveness, its widespread clinical application is limited by its cost, testing duration, and examiner experience. Alternative vision measures that are quicker and more straightforward, while remaining repeatable and sensitive to clinical change, are required.

The study aims to assess the utility of LLVA and LLD as clinical markers of central retinal function in two distinct inherited retinal diseases: choroideremia and *RPGR*-associated RP. We hypothesize that LLVA and LLD are more sensitive markers of impaired central visual function than standard VA in both conditions. In addition, we hope to learn more about the physiologic function underpinning LLVA.

## Methods

Patients with choroideremia and *RPGR-*associated RP were assessed as part of the screening process, prior to recruitment into gene therapy trials (NCT02407678 and NCT03116113), at Oxford Eye Hospital, in accordance with the tenets of the Declaration of Helsinki. Control data came from the Electronic VA study (UK Health Regulatory Authority reference 17/NS/0036).^21^ Patients with very advanced retinal disease stages unable to resolve any high-contrast VA letters were excluded, as well as those with copathologies such as diabetic retinopathy, glaucoma, or other ocular disease.

### Visual Acuity Testing

All tests were completed in standard clinical trial conditions, with the room lights switched off, using a retro-illuminated (l60 cd/m^2^) ETDRS chart (Precision Vision, Bloomington, IL, USA).^22^ LLVA was assessed first using the standard high-contrast ETDRS chart, placed at 4 m or 1 m, and a 2.0 log unit neutral density filter was placed in front of the testing eye. The participants were instructed to read down the chart until they could not accurately resolve any more letters. There was no formal dark adaptation attempted. To minimize the possibility of participants memorizing the letters, LLVA was tested first (before standard VA) as the participants read fewer letters under these conditions. The same procedure, without the neutral density filter, was repeated for standard VA assessment.

### Visual Acuity Test-Retest Variability

A subset of patients with choroideremia and *RPGR*-associated RP (*n* = 19) and control participants (*n* = 18) underwent repeat LLVA and standard VA testing within 2 weeks of the initial visit to assess repeatability. The same room setup was used, and the tests were conducted in exactly the same manner.

### Microperimetry

Central retinal sensitivity measurement using MAIA microperimetry (Centervue SpA, Padova, Italy) was performed on all patients with choroideremia and *RPGR*-associated RP without any formal dark adaption.^23^ A standard 10-2 test grid was used, with a 4-2 bracketing threshold strategy and Goldmann size III stimulus of various intensities presented on a mesopic background (4 apostilbs). Tests were judged as reliable if the fixation losses were 20% or less, corresponding to less than 20% positive catch trials presented to the patient's physiological blind spot; any unreliable results were repeated.

### Retinal Imaging

Optical coherence tomography (OCT) volume scans of participant groups were taken using Heidelberg Eye Explorer software (Heidelberg Engineering, Heidelberg, Germany). A horizontal line scan through the foveal pit was selected and the OCT software built-in caliper used to measure the ellipsoid zone width in micrometers (µm).^24^ The clinical fovea was defined as up to 400 µm representing 0.5–1.0 degrees of central vision, and the clinical macula was defined as up to 1500 µm, representing 3–5 degrees of central vision.^25^

### Statistical Analysis

Statistical analysis was performed using SPSS (version 25; IBM Software, New York, NY, USA) and figures created in Sigma Plot (version 14; Systat Software, San Jose, CA, USA). Due to the nonnormal distribution of the data, nonparametric comparative statistical tests were applied with Bonferroni-adjusted significances where required. For repeatability, Bland-Altman analyses were used.^26^ The VA units included the number of ETDRS letters seen. Microperimetry indices included mean sensitivity in decibels (dB). Analyses of the right eye only were performed to reduce statistical errors from using highly correlated data from both eyes.^27^ For a LLD, subanalyses of both the *RPGR*-associated RP and choroideremia patient sets were split into two further subgroups; group 1 (low LLD) consisted of those with 13 or fewer LLD letters, while group 2 (high LLD) had a LLD of more than 13 letters. A LLD above 13 was selected as the cutoff as it was the upper quartile of the control group and is the upper LLD normal limit defined by a meta-analysis of 130 healthy controls in a recent literature review.^18^

## Results

Forty-three participants with a confirmed *CHM* pathogenic variant, 24 participants with a confirmed *RPGR* pathogenic variant, and 62 healthy controls were assessed. All three groups were age-matched (Kruskal-Wallis test, *P* = 0.413). The results show both standard VA and LLVA (Table 1) are significantly reduced in both *RPGR*-associated RP and choroideremia compared to controls (Kruskal-Wallis test with post hoc analyses, *P* ≤ 0.01). Once the participant groups were divided into low (≤13) and high (>13) LLD groups, standard VA showed no statistically significant difference between the high and low LLD groups in both *RPGR*-associated RP and choroideremia patient sets (Mann-Whitney *U* test, *P* = 0.392 and *P* = 0.113, respectively). Microperimetry mean sensitivity was significantly reduced in the high LLD subgroups compared with low LLD subgroups in both choroideremia (Mann-Whitney *U* test, *P* = 0.001) and *RPGR*-associated RP (Mann-Whitney *U* test, *P* = 0.001). Similarly, the OCT ellipsoid zone width was also significantly reduced in both choroideremia and *RPGR-*associated RP in high LLD subgroups compared to low LLD subgroups (Mann-Whitney *U* test, *P* = 0.02 and *P* = 0.04, respectively). This indicates that while standard VA remained relatively unaffected, the LLVA was significantly impaired and appeared associated with both structural (OCT ellipsoid zone width) and functional (microperimetry sensitivity) markers.

**Table 1.** Participant Age-Matched Demographics and VA Results for All Three Participant Groups, With *RPGR*-Associated RP and Choroideremia Divided into Two LLD Subgroups

Characteristic	*n*	Age, y	Median Standard VA, ETDRS Letters (IQR)	Median LLVA, ETDRS Letters (IQR)	Median LLD, ETDRS Letters (IQR)	Median Central Retinal Sensitivity, dB (IQR)	OCT Ellipsoid Zone Width, µm (IQR)
*RPGR*-associated RP							
Group 1 (low LLD, ≤13)	13	31 (26–51)	68 (63–77)	59 (50–68)	12 (8–12)	3.6 (1.3–4.7)	836^a^ (520–1311)
Group 2 (high LLD, >13)	11	32 (26–49)	72 (33–75)	9 (0–55)	30 (19–52)	0.0 (0.0–0.5)	0 (0–358)
Total data set	24	32 (26–51)	70 (57–75)	52 (5–61)	13 (11–26)	1.2 (0.0–4.0)	480^a^ (0–964)
Choroideremia							
Group 1 (low LLD ≤13)	16	32 (22–45)	78 (71–81)	66 (61–76)	10 (7–12)	8.5 (4.5–14.8)	2114 (967–3366)
Group 2 (high LLD >13)	27	46 (35–58)	68 (60–82)	43 (29–64)	21 (17–40)	1.0 (0.0–3.6)	596 (303–1661)
Total data set	43	44 (31–53)	74 (65–82)	58 (38–69)	16(12–22)	3.0 (0.7–9.5)	935 (509–2352)
Controls	62	36 (30–51)	88 (84–94)	79 (73–82)	11 (7–13)	—	—

Group 1 (low LLD): participants with 13 or fewer low LLD. Group 2 (high LLD): participants with more than 13 LLD.

a One RPGR participant did not have OCT imaging; *RPGR* group 1, *n* = 12. Total *n* = 23.

**Table 2.** Coefficient of Repeatability and Limits of Agreement for Both Standard VA and LLVA in Healthy Controls and Combined Rod-Cone Degeneration Group Comprising Patients with Choroideremia and *RPGR*-Associated RP

Group	*n*	Standard VA Coefficient of Repeatability (CoR)	LLVA CoR
Controls, ETDRS letters limits of agreement (LoA)	18	7.24 (+6.55 to −7.99)	5.97 (+5.02 to −6.91)
Rod-cone degeneration, ETDRS letters (LoA)	19	7.23 (+9.12 to −5.34)	6.69 (+6.80 to −6.59)

### Test-Retest Variability

The coefficients of repeatability for LLVA were equivalent to 0.12 LogMAR (six ETDRS letters) and 0.14 LogMAR (seven ETDRS letters) for controls and the rod-cone degenerative group, respectively (Table 2). This compares favorably to the standard VA repeatability, 0.14 LogMAR, measured in both groups and is within the accepted VA repeatability.

### LLVA and Standard VA Correlation Analysis

**Figure 1.**
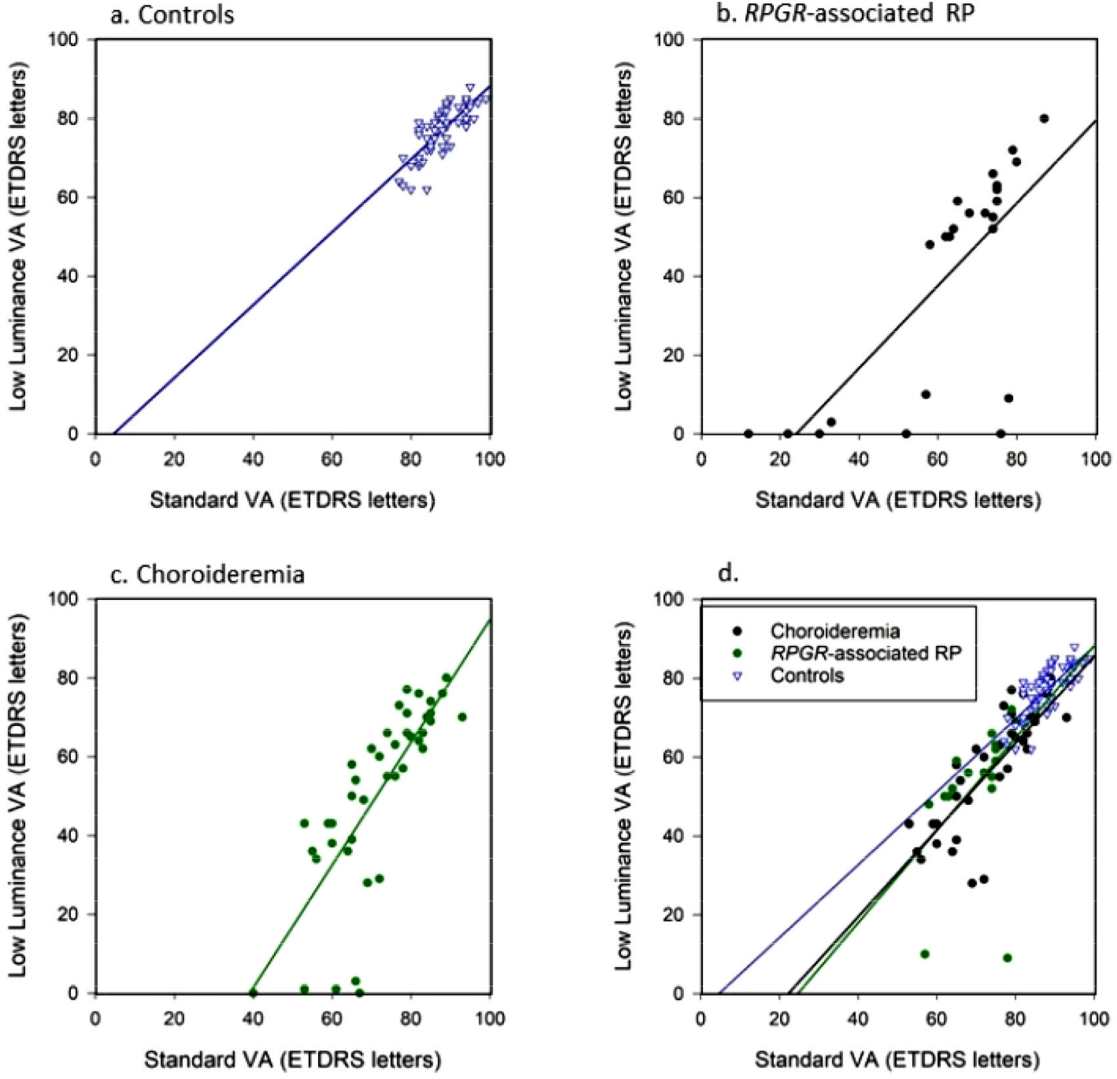
Spearman rank significant positive correlations between standard VA and LLVA: (a) controls (ρ = 0.81, *P* < 0.001), (b) patients with *RPGR*-associated RP (ρ = 0.72, *P* < 0.001), and (c) patients with choroideremia (ρ = 0.85, *P* < 0.001). (d) Subanalysis after choroideremia and *RPGR*-associated RP zero LLVA values are removed. The *blue trend line* represents controls, the *solid black trend line* represents RPGR-associated RP, and the *dotted green line* represents the choroideremia trend line.

In all three participant groups, LLVA correlated significantly with standard VA (Fig. 1). Interestingly, 11 participants in both choroideremia (*n* = 5) and *RPGR*-associated RP sets (*n* = 6) demonstrated very poor or nonexistent LLVA (≤3 ETDRS letters) despite relatively well-preserved standard VA, resulting in a very high LLD result. This effect was observed in patients with choroideremia once LLVA reduced below approximately 35 ETDRS letters and in patients with *RPGR*-associated RP below approximately 50 ETDRS letters.

**Figure 2.**
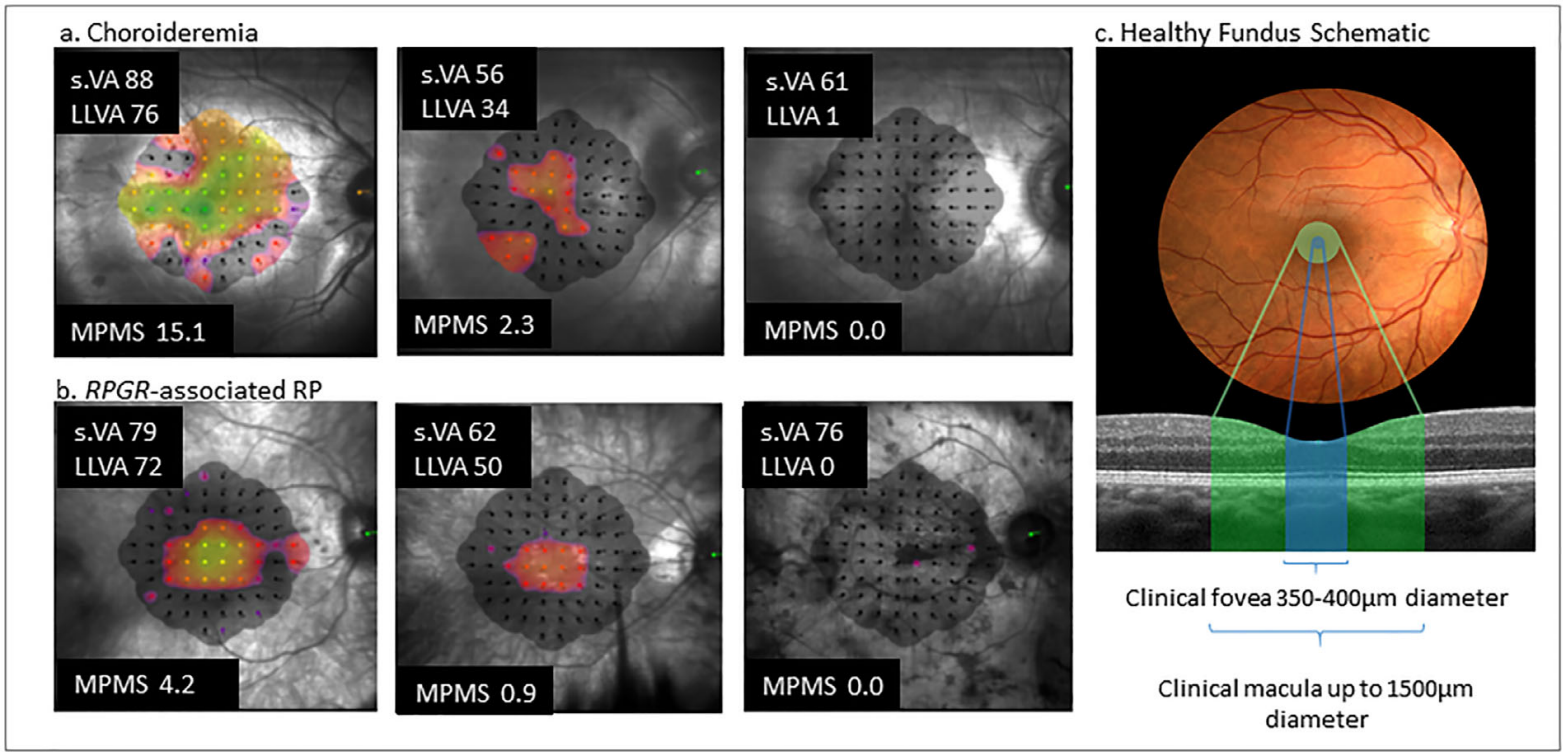
(a) Microperimetry plot results, standard VA (s.VA) and LLVA for three patients with choroideremia with a range of central retinal sensitivity function to represent different disease stages. The brighter color the threshold sensitivity map, the greater the central function; the *darker red*, *purple*, and black areas indicate reducing sensitivity function. (b) *RPGR*-associated RP microperimetry results with standard VA and LLVA of three patients with a range of central retinal sensitivity function representing different disease stages. (c) A schematic diagram to illustrate the clinical definitions of the extent of the foveal and macular areas. MPMS, microperimetry mean sensitivity.

To investigate the standard VA and LLVA correlation prior to the LLVA floor effects, the LLVA zero values were removed from the choroideremia (*n* = 5 removed) and *RPGR*-associated RP (*n* = 6 removed) data sets and the correlations recalculated (Fig. 2d). In all three groups, the correlation between LLVA and standard VA remained highly significant (controls: ρ = 0.81, *P* < 0.001; choroideremia: ρ = 0.84, *P* < 0.001; and *RGPR*-associated RP: ρ = 0.70, *P* = 0.001). There was no significant difference between the correlation coefficient for both controls and choroideremia (Steiger's *z* test: *z* = 0.44, *P* = 0.65) and between controls and *RPGR*-associated RP (Steiger's *z* test: *z* = 0.13, *P* = 0.37). This indicates at earlier disease stages (shown by better VAs), standard VA and LLVA deteriorate at a similar rate, and so the LLD remains low.

### LLD and Retinal Function and Structure Relationships

To investigate the sudden drop in LLVA function in both disease groups (Figs. 1b, 1c), we analyzed the LLD structure-function correlations and hypothesized that in the presence of preserved standard VA, LLVA (and subsequently LLD) is dependent on the area or sensitivity of central retinal function. Figure 2 illustrates examples of patients from Table 1, those with a small LLD (preserved LLVA) and greater central retinal sensitivity alongside those with a larger LLD (reduced LLVA) and reduced central retinal sensitivity.

**Figure 3.**
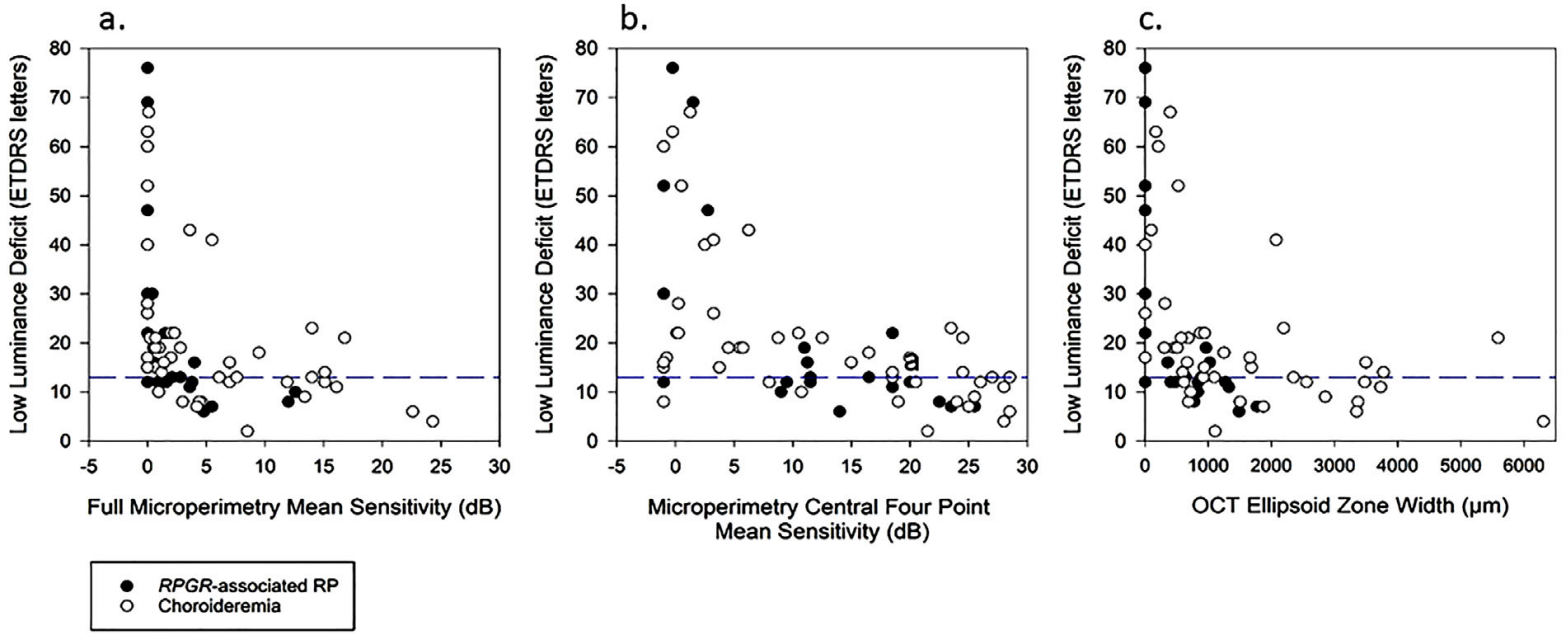
The relationships between LLD and structure-function variables such as OCT and microperimetry. (a) Microperimetry mean sensitivity and LLD showing a significant negative Spearman rank correlation for both choroideremia (ρ = −0.59, *P* < 0.001) and *RPGR*-associated RP (ρ = −0.83, *P* < 0.001). (b) Low luminance deficit and microperimetry central 4-point mean sensitivity correlation analysis, showing a significant negative Spearman rank correlation again with both choroideremia (ρ = −0.61, *P* < 0.001) and *RPGR*-associated RP (ρ = −0.68, *P* < 0.001). (c) OCT ellipsoid zone width and LLD correlation, with both data sets showed a significant Spearman rank correlation, choroideremia (ρ = −0.59, *P* < 0.001) and *RPGR*-associated RP (ρ = −0.76, *P* < 0.001). The horizontal *blue dashed lines* highlight 13 LLD, which is the upper normal limit for controls.

There was a significant negative correlation with LLD and microperimetry (Fig. 3a). The LLD increased dramatically at very low full microperimetry mean sensitivities, indicating relatively good standard VA in the presence of little or no LLVA. To further focus analyses on foveal function, the mean sensitivity of the central four points (those closest to the fovea) of the microperimetry 10-2 plot were calculated (Fig. 3b). This shows a more gradual yet still significant negative Spearman rank correlation; LLD increased gradually with deteriorating central four point mean sensitivity, before increasing more dramatically with less than 5 dB central mean sensitivity. While standard VA was preserved, the LLVA fell as the microperimetry central sensitivity dropped, increasing the LLD values.

To compare retinal structure changes, the OCT ellipsoid zone width was correlated with the LLD. Figure 3c shows where LLD increased steadily with decreasing ellipsoid width, before LLD increased dramatically as ellipsoid widths became less than 500 µm, within the boundaries of the clinical foveal area (Fig. 2c). In these cases, standard VA was preserved and LLVA was reducing, causing the increased LLD. Those with LLD within normal limits (preserved standard VA and relatively preserved LLVA function) tended to show wider ellipsoid zone widths, extending beyond the clinical foveal area and into the clinical macular area.

LLVA function appears dependent on a minimal level of central sensitivity and width of the ellipsoid zone. Those 11 patients with very poor LLVA scores (Figs. 1b, 1c) had no detectable central microperimetry sensitivity and very constricted, some unmeasurable, ellipsoid zone widths (median, 0 µm; interquartile range [IQR], 0–189.5 µm) despite more reasonable standard VA (median, 52 ETDRS letters; IQR, 31.5–63.5).

## Discussion

In *RPGR*-associated RP and choroideremia, LLVA is adversely affected prior to the loss of standard VA. LLVA is quick to perform and can be undertaken with standard ophthalmic equipment. It has equivalent repeatability to standard VA.^28^^,^^29^ The choroideremia and *RPGR-*associated RP results compare to previously reported LLVA repeatability values (±0.10 to ±0.15 logMAR) found in healthy and age-related macular degeneration participants.^30^^,^^31^

Figure 1d shows that in earlier disease stages, LLVA progression is similar to standard VA. Changes in LLVA measures only become significant in more moderate to advanced disease, in which a dissociation between LLVA and standard VA arises. Here this dissociation is described by an increasing LLD. In those patients with relatively preserved standard VA but very poor LLVA (high LLD), this marks the beginning of end-stage disease (illustrated by Fig. 2), prior to the eventual loss in standard VA. We believe these effects occurred earlier in *RPGR*-associated RP (around 35 ETDRS letters standard VA) than in choroideremia (around 50 standard ETDRS letters standard VA) due to the mechanisms of the disease.

In *RPGR-*associated RP, the photoreceptors are primarily affected, whereas in choroideremia, the retinal pigment epithelium is affected first. LLVA is a marker of central cone function.^14^^,^^32^ If the remaining island of cone photoreceptor function is reduced in earlier disease stages in *RPGR-*associated RP, as reported by Menghini et al.,^33^ this could explain the earlier drop in LLVA, in contrast to choroideremia, in which the central cone function is more preserved until later disease stages,^6^ reflected by the significantly higher microperimetry mean sensitivities compared to *RPGR*-associated RP (Table 1).

LLD is a frequently reported outcome measure as it highlights the level of dissociation between standard VA and LLVA.^14^^,^^16^ However, there are situations in which the raw LLVA is useful, as it can reveal the extent of foveal function. A significant example of this was reported with geographic atrophy following foveal involvement and subsequent reduction of standard VA.^14^ A patient with geographic atrophy may have a standard VA of 35 letters due to compromised central foveal function and a LLVA of 30 letters due to intact peripheral foveal function. This produces a “normal” low LLD of 5 letters despite clearly reduced central function—hence our decision to report both LLVA and LLD in this work. Neither measure should be interpreted without reference to the standard VA.

Molina-Martín et al.^34^ reported a median central retinal sensitivity value of 32.9 dB (IQR, 1.8) in 237 healthy participants, which is significantly higher than the median central retinal sensitivity values for our patients with choroideremia and *RPGR*-associated RP (Table 1). However, the MAIA considers “normal” sensitivity to be >25 dB, but further investigation is needed to account for age and retinal loci sensitivity variation.^35^ Those patients with poorer mean central retinal sensitivities and smaller ellipsoid zone widths tended to have reduced LLVA (and subsequently a larger LLD). In contrast, those with a small LLD and preserved LLVA were associated with better central retinal sensitivity and a wider ellipsoid zone width that extended into and beyond the clinical macula (Fig. 3). Due to the centripetal nature of the degeneration seen in these two rod-cone degenerations, especially *RPGR*-associated RP as it is more symmetrical than choroideremia, LLVA function (and a low LLD) is more dependent on a larger area of preserved central macular function than standard VA. This supports of our hypothesis that LLVA represents a greater degree of the clinical macular region and therefore makes a useful marker of clinical macular function.

Microperimetry is emerging as a key assessment of central retinal function for retinal disease.^36^ However, it requires expensive equipment, is lengthy to perform, and requires skilled examiners. From a patient perspective, it can be very tiring. A patient with no LLVA function had no measurable central retinal sensitivity with 10-2 microperimetry; LLVA could therefore make a useful screening tool to determine whether 10-2 microperimetry testing is warranted in these patient groups (Figs. 2, 3).

Blurred photoreceptor margins limit ellipsoid zone analyses. To reduce measurement variability, two examiners reviewed several images twice (LJW and TMWB). In advanced choroideremia disease stages, often the surviving visual islands are asymmetrical and in a nonfoveal retinal location. Retinal structure, including rod-cone distribution and size of postreceptoral units, changes with eccentricity.^37^ Therefore, visual island asymmetry and location may increase LLVA (and LLD) variability. Furthermore, the ellipsoid zone width is not a comprehensive indicator of photoreceptor function; some photoreceptors within the ellipsoid zone may be functioning poorly, possibility due to a shortened outer segment.^33^ Other structural markers, such as the area of the autofluorescence islands in choroideremia and the diameter to the hyperautofluorescent ring in *RPGR*-associated RP, have been shown to be useful disease markers.^38^^,^^39^ However, these autofluorescence patterns in these two conditions are not directly comparable.

The underlying morphologic mechanisms behind LLVA, however, remain unclear. The larger area of preserved central macula required for LLVA could be linked to the hypothesized circuit function of horizontal and amacrine cells within the plexiform layers, creating greater low light sensitivity.^40^

## Conclusion

Both LLVA and LLD reflect different visual functions but together are useful earlier clinical markers of central retinal function in choroideremia and *RGPR-*associated RP, in which standard VA is preserved. Standard VA, LLVA, and LLD are interlinked and should be interpreted in unison. While standard VA reflects clinical foveal function, LLVA appears to reflect the function of a greater area of macular sensitivity. The LLD enables overall interpretation of the relationship between standard VA and LLVA and subsequent central retinal integrity. In choroideremia and *RGPR-*associated RP, a severely reduced or no LLVA but good standard VA (large LLD) indicates that the patient is at the beginning of end-stage disease, and loss of foveal function is impending. This may be of use in guiding patient prognosis. A small LLD is a marker for the likely presence of central retinal sensitivity detectable with standard 10-2 microperimetry and may be able to screen patient suitability for subsequent microperimetry assessment. We believe these findings are likely to extrapolate to other rod-cone degenerations, but further investigation is required.
